# Supplementary material for: Gaze-in-wild: A dataset for studying eye and head coordination in everyday activities
Source: Sci Rep. 2020 Feb 13;10:2539. doi: 10.1038/s41598-020-59251-5 (PMC7018838; doi:10.1038/s41598-020-59251-5)
Supplement: Supplementary file 1 — Supplementary Information. [file 41598_2020_59251_MOESM1_ESM.pdf]

**Manuscript Title**: Gaze-in-wild: A dataset for studying eye and head coordination in everyday activities

**Author list**: Rakshit Kothari, Zhizhuo Yang, Christopher Kanan, Reynold Bailey, Jeff Pelz, Gabriel Diaz

| Person | Age | Indoor navigation | | | Ball catching | | | Visual search | | | Tea making | | |
|---|---|---|---|---|---|---|---|---|---|---|---|---|---|
| | | Status | $\Delta_\theta^{ETG}$ | $\Delta_\theta^{IMU}$ | Status | $\Delta_\theta^{ETG}$ | $\Delta_\theta^{IMU}$ | Status | $\Delta_\theta^{ETG}$ | $\Delta_\theta^{IMU}$ | Status | $\Delta_\theta^{ETG}$ | $\Delta_\theta^{IMU}$ |
| 1 | 27 | $\surd\surd$ | 0.75 | 8.71 | $\surd\surd$ | 0.67 | 7.90 | $\times$ | | | $\times$ | | |
| 2 | 18 | $\overset{+}{\surd\surd}$ | 0.79 | 0.76 | $\overset{+}{\surd\surd}$ | 0.89 | $6.63^{\gamma}$ | $\times$ | | | $\surd$ | 0.76 | |
| 3 | 19 | $\surd$ | 1.27 | 0.05 | $\overset{+}{\surd}$ | 0.75 | 2.41 | $\times$ | | | $\times$ | | |
| 6 | 18 | $\surd$ | 0.43 | $45.01^{\gamma}$ | $\surd\surd$ | 0.37 | $10.11^{\gamma}$ | $\times$ | | | $\overset{+}{O}$ | 0.69 | 0.84 |
| 8 | 22 | $\surd$ | 0.99 | 4.67 | $\surd$ | 1.29 | 1.91 | $\surd$ | 0.98 | 0.56 | $\times$ | | |
| 9 | 22 | $\surd$ | 0.62 | $3.66^{\gamma}$ | $\surd$ | 0.73 | 2.95 | $O$ | 0.59 | 0.27 | $\times$ | | |
| 10 | 26 | $\surd$ | 0.55 | 13.00 | $O$ | 1.29 | 7.36 | $O$ | 1.04 | 0.01 | $O$ | 0.49 | 0.73 |
| 11 | 25 | $O$ | 0.77 | $31.96^{\gamma}$ | $O$ | 0.56 | 4.03 | $\times$ | | | $O$ | 0.69 | 4.63 |
| 12 | 26 | $\surd$ | 0.52 | 2.83 | $\surd$ | 0.65 | 2.04 | $\surd$ | 0.75 | 1.17 | $\surd$ | 0.57 | 12.17 |
| 13 | 27 | $O$ | 1.14 | 3.70 | $O$ | 1.08 | 0.44 | $O$ | 0.83 | 7.52 | $O$ | 0.80 | 14.14 |
| 14 | 23 | $O$ | 0.88 | 0.34 | $O$ | 1.20 | 0.02 | $O$ | 0.82 | 1.63 | $O$ | 1.08 | 1.19 |
| 15 | 22 | $O$ | 1.09 | 8.33 | $O$ | 0.90 | 1.33 | $\surd$ | 0.73 | $15.56^{\gamma}$ | $O$ | 0.67 | 7.43 |
| 16 | 22 | $\surd$ | 0.82 | 0.41 | $\surd$ | 0.55 | 0.53 | $O$ | 0.94 | 5.31 | $O$ | 0.92 | 0.88 |
| 17 | 23 | $\surd$ | 1.01 | 4.28 | $\surd$ | 0.89 | 0.22 | $\surd$ | 0.65 | 0.22 | $O$ | 0.79 | $22.76^{\gamma}$ |
| 18 | 34 | $\surd$ | 1.01 | 14.46 | $O$ | 0.99 | 1.63 | $\surd$ | 0.71 | 12.09 | $\surd$ | 0.81 | 3.07 |
| 19 | 26 | $O$ | 0.88 | 2.38 | $\surd$ | 0.73 | 6.52 | $\surd$ | 0.96 | $19.34^{\gamma}$ | $\times$ | | |
| 20 | 55 | $\surd$ | 0.93 | 7.01 | $O$ | 1.23 | 3.58 | $\surd$ | 0.95 | $20.47^{\gamma}$ | $O$ | 1.24 | 2.87 |
| 22 | 54 | $\surd$ | 0.50 | 6.84 | $\surd$ | 0.38 | 6.89 | $\surd$ | 0.55 | 11.61 | $\times$ | | |
| 23 | 60 | $O$ | 1.23 | 7.45 | $O$ | 0.47 | 3.50 | $O$ | 0.44 | 2.04 | $O$ | 0.44 | 2.54 |

**Table 1.** Dataset status and error measures. $\Delta_\Theta^{ETG} \rightarrow$ Calibration error in degrees $^\circ$. $\Delta_\Theta^{IMU} \rightarrow$ IMU absolute angular drift measured by the difference in the head orientation at the start and end of a task in $^\circ$. Note that certain recordings do not have this measure because we did not explicitly constrain the participants to arrive at their initial pose. For such situations, we estimate head pose when subjects were approximately fixated towards an identifiable direction using visual imagery in the middle of a task. Status symbols: $\surd\surd \rightarrow$ recording has labels from multiple labellers. $\surd \rightarrow$ recording is labeled by a single person. $O \rightarrow$ Not labeled. $+ \rightarrow$ Depth values not present. $\gamma \rightarrow$ Manual head pose correction. $\times \rightarrow$ Data discarded.

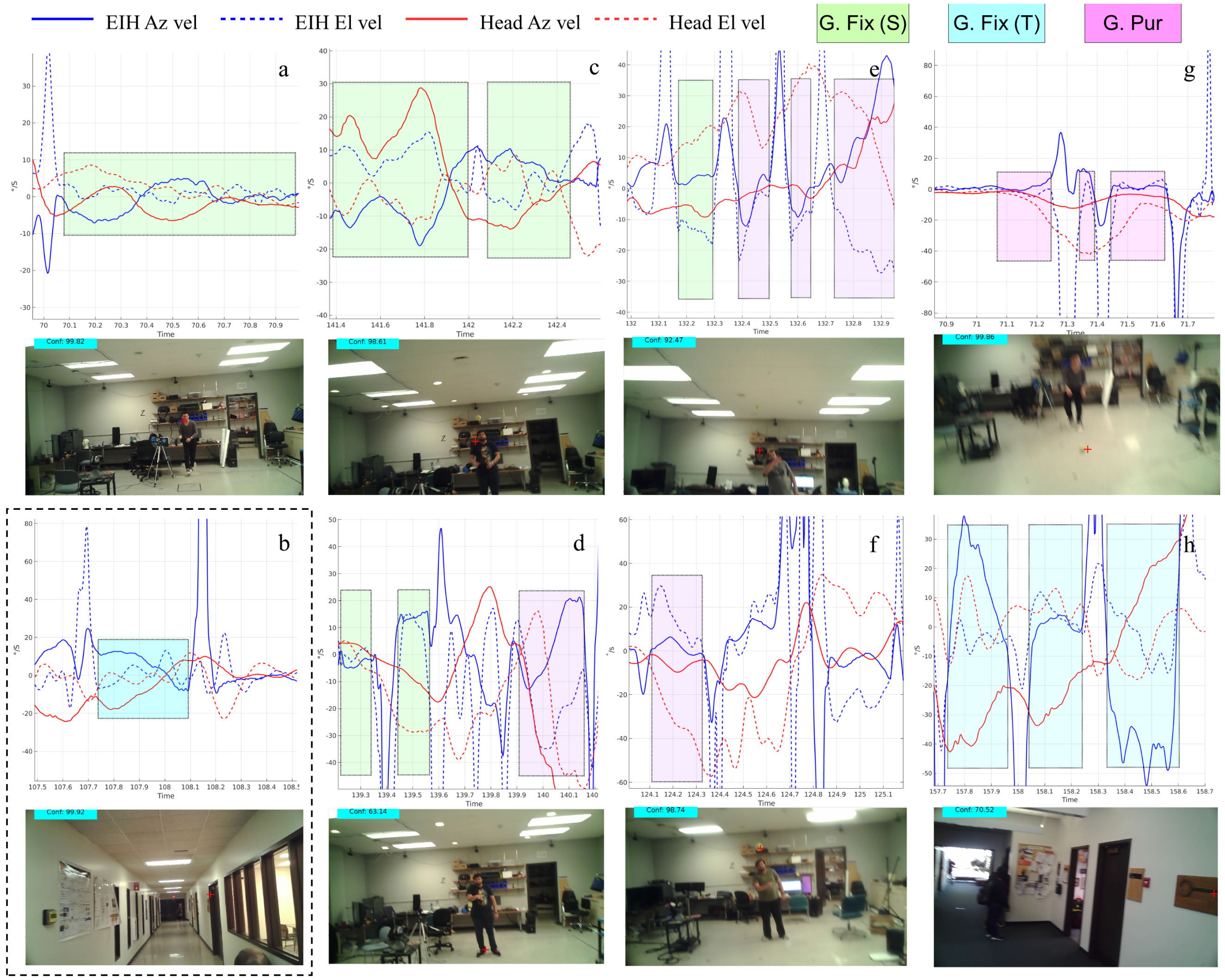


**Figure 1.** Examples of gaze behavior in $1s$ clips. Eye and head velocity signals and the related world camera view which best describes the signals is provided as a pair in each row. The bold blue line signifies EiH azimuthal velocity while the dashed line signifies EiH elevation velocity. Likewise, the red variant stands for head velocity traces. Rectangular boxes within each sub-figure are used to draw the readers attention to marked events. Sub-figure **a** and **b** signify gaze fixation events. Note that due to a slight misalignment in the IMU and eye tracker, the velocity components in **a** do not precisely cancel out. Sub-figure **c** and **d** showcase gaze fixation events while the head catches up to the fixation location. Notice that in **d**, the EiH and head velocity traces do not cancel out despite being marked as a fixation. Signal information alone convey that the traces are a head dominated pursuit movement, however, the world imagery shows that the head is catching up to the fixation location. Sub-figures **e**, **f** and **g** highlight examples of head dominated pursuit behavior punctuated by saccades. Note the fixation event marked in **e**. It appears that the eye and head signals compensate in the elevation component but head movements dominate in the azimuthal direction. In hindsight, it appears to be part of a sequence of pursuit events interjected by predictive saccades but has been incorrectly marked as a fixation. Sub-figure **h** is an interesting example of fixation under translation. Visual imagery conveys that the underlying behavior is fixational but one can see that the velocity traces resembles that of a pursuit movement. Examples listed in **d** and **h** contribute heavily to fixation/pursuit confusion.

| | Metric | biRNN | biRNN (only eyes) | biRNN (only absolute) | fRNN | RF |
|---|---|---|---|---|---|---|
| Sample level metrics | Overall $\kappa$ | 0.61 | 0.56 | 0.58 | 0.54 | 0.63 |
| | G. Fix $\kappa$ | 0.61 | 0.55 | 0.57 | 0.54 | 0.63 |
| | G. Pur $\kappa$ | 0.37 | 0.24 | 0.33 | 0.29 | 0.28 |
| | Sac $\kappa$ | 0.69 | 0.71 | 0.71 | 0.68 | 0.74 |
| Event level metrics | Overall ($\kappa$*, $\kappa$) | 0.37*, 0.47 | 0.38*, 0.44 | 0.40*, 0.47 | 0.27*, 0.35 | 0.37*, 0.32 |
| | G. Fix ($\kappa$*, $\kappa$) | 0.34*, 0.61 | 0.32*, 0.56 | 0.36*, 0.59 | 0.21*, 0.49 | 0.31*, 0.46 |
| | G. Pur ($\kappa$*, $\kappa$) | 0.14*, 0.27 | 0.08*, 0.06 | 0.17*, 0.28 | 0.03*, 0.12 | 0.03*, 0.01 |
| | Sac ($\kappa$*, $\kappa$) | 0.44*, 0.67 | 0.52*, 0.71 | 0.51*, 0.70 | 0.44*, 0.63 | 0.54*, 0.63 |
| | G. Fix $F_1$ | 0.80 | 0.79 | 0.80 | 0.74 | 0.74 |
| | G. Pur $F_1$ | 0.35 | 0.25 | 0.34 | 0.22 | 0.26 |
| | Sac $F_1$ | 0.83 | 0.85 | 0.85 | 0.81 | 0.82 |
| | G. Fix $l_2$ ($\mu$, $\sigma$) | 14.93, 3.64 | 15.11, 3.46 | 15.13, 3.65 | 15.09, 3.32 | 12.97, 2.10 |
| | G. Pur $l_2$ ($\mu$, $\sigma$) | 14.25, 3.84 | 13.34, 3.75 | 12.99, 4.90 | 15.70, 3.44 | 15.15, 3.44 |
| | Sac $l_2$ ($\mu$, $\sigma$) | 14.73, 3.62 | 14.65, 3.52 | 14.87, 3.73 | 15.01, 3.42 | 12.80, 2.16 |
| | G. Fix $O_r$ | 0.92 | 0.91 | 0.91 | 0.90 | 0.93 |
| | G. Pur $O_r$ | 0.90 | 0.91 | 0.91 | 0.89 | 0.88 |
| | Sac $O_r$ | 0.71 | 0.71 | 0.71 | 0.69 | 0.75 |
| | G. Fix *de* | 0.21 | 0.20 | 0.18 | 0.14 | 0.09 |
| | G. Pur *de* | 0.20 | 0.06 | 0.14 | 0.13 | 0.02 |
| | Sac *de* | 0.05 | 0.05 | 0.06 | 0.06 | 0.11 |

**Table 2.** Ablation results reported using all metrics. Note that event level $\kappa$* scores and ratio of detached events (*de*) are computed using the ELC matching technique as described in Section 4. Mean $l_2$ distance and it's deviation, $l_2 - \sigma$ between between matched events are reported in *ms*.
